# Quantitative EEG and Cognitive Decline in Parkinson's Disease

**DOI:** 10.1155/2016/9060649

**Published:** 2016-04-11

**Authors:** Vitalii V. Cozac, Ute Gschwandtner, Florian Hatz, Martin Hardmeier, Stephan Rüegg, Peter Fuhr

**Affiliations:** Universitätsspital Basel, Abteilung Neurophysiologie, Petersgraben 4, 4031 Basel, Switzerland

## Abstract

Cognitive decline is common with the progression of Parkinson's disease (PD). Different candidate biomarkers are currently studied for the risk of dementia in PD. Several studies have shown that quantitative EEG (QEEG) is a promising predictor of PD-related cognitive decline. In this paper we briefly outline the basics of QEEG analysis and analyze the recent publications addressing the predictive value of QEEG in the context of cognitive decline in PD. The MEDLINE database was searched for relevant publications from January 01, 2005, to March 02, 2015. Twenty-four studies reported QEEG findings in various cognitive states in PD. Spectral and connectivity markers of QEEG could help to discriminate between PD patients with different level of cognitive decline. QEEG variables correlate with tools for cognitive assessment over time and are associated with significant hazard ratios to predict PD-related dementia. QEEG analysis shows high test-retest reliability and avoids learning effects associated with some neuropsychological testing; it is noninvasive and relatively easy to repeat.

## 1. Introduction

*(1) Background.* Cognitive decline is common with the progression of Parkinson's disease (PD) [1]. Several studies have shown that the point prevalence of dementia in patients with PD (PD-D) is about 30% and that the incidence rate of dementia in PD is 4–6 times higher than in control subjects [2–4]. The cumulative prevalence of PD-D in patients surviving more than ten years after diagnosis was estimated at more than 75% [5]. Thus, prediction and early diagnosis of cognitive decline in PD are a current challenge in neurosciences as well as patient care and counselling. Various markers have been studied for early identification of PD-D and mild cognitive impairment related to PD (PD-MCI) [6–8]. Quantitative EEG (QEEG) has shown good potential in identification of cognitive deterioration in patients with PD [9]. QEEG is advancing fast, and various new methods have been introduced and applied in QEEG research. In this review, we briefly discuss the basics of QEEG and recent publications addressing its predictive value for detecting of PD-related worsening of cognition.

*(2) Methods of Literature Search*. References for this review were identified through search of the MEDLINE database (Supplement 1 in Supplementary Material available online at http://dx.doi.org/10.1155/2016/9060649). The following search strategy was used: ((((eeg) AND parkin^*∗*^))) AND (“2005” [Date - Publication]: “2015” [Date - Publication]). We identified 739 potentially eligible publications with this search query on March 2, 2015. The titles and abstracts were examined for selection criteria:

- full text available in English;
- original research studies;
- subjects of the study: patients with PD, who were assessed by QEEG (spectral or/and connectivity analysis) and had not undergone deep brain stimulation;
- QEEG variables acquired through conventional EEG machines or magnetoencephalography (MEG) in resting state eyes-closed conditions in “ON”* or/and* “OFF” levodopa medication condition;
- studies focusing on comparison between groups of PD patients with different states of cognition (e.g., PD-D versus PD-MCI)* or/and* longitudinal QEEG evaluations of cognition in patients with PD* or/and* evaluations of correlation of QEEG variables with tests and tools for cognitive assessment.

Sixty-one original research papers were identified after analysis of the titles and abstracts and subject to full text analysis. After analysis of the full text, 23 original research publications in peer-reviewed journals were selected for the final analysis. Details summarizing the profiles of the included publications are shown in Table 1. Profiles of the excluded papers are shown in Supplement 2.

*(3) Analysis of the Findings.* These 23 selected studies were performed by nine independent research groups. Independence of the authors was analyzed by reviewing the affiliations of the first and the corresponding authors.

Full meta-analysis was not performed because of the following reasons: firstly, in spite of a common concept, applying QEEG methods to investigate cognition of patients with PD, these studies were too heterogeneous in terms of the applied methods. The researchers use different methods of mathematical processing of the EEG, different approaches (such as spectral or connectivity analysis), and different settings. Secondly, while there is a more or less common consensus regarding diagnostic criteria of an advanced cognitive deterioration, PD-dementia (PD-D), such a consensus regarding diagnostic criteria for intermediate (between normal cognition and PD-D) cognitive disorder, mild cognitive impairment (MCI), is still under discussion [10–12].

However, the effect sizes of the reported variables were calculated in order to compare the relevant results. The effect size is a statistical measure, reflecting how much two standardized means are different between two populations [13]. The larger the effect size is, the more the two populations are distinct in a studied parameter. Similarly, correlation coefficients were analyzed by Fisher's *Z* transformation [14]. In this case, the larger the Fisher *Z* is, the stronger the correlation is.

## 2. Background on QEEG

### 2.1. Basics of Quantitative Analysis of EEG

**Figure 1:**
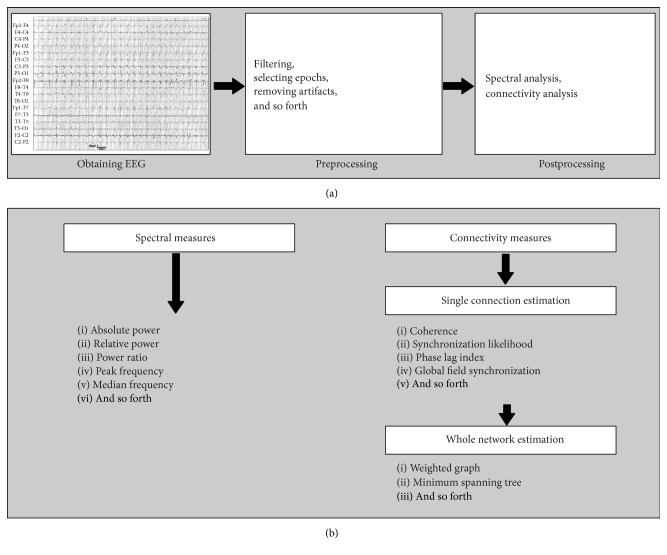
Outlines of the QEEG process. (a) Main steps of the processing; (b) spectral and functional connectivity measures.

QEEG is a mathematical processing of EEG data to extract relevant information for subsequent analysis or comparison with other kinds of data [15, 16]. In contrast to conventional EEG, where electrical activity of the brain cells is visually analyzed, QEEG provides derivative parameters, which are generated from EEG “raw” data using computational methods. QEEG includes several procedural steps (Figure 1). The first step consists of* EEG signal acquisition* itself, performed with the use of various EEG machines and electrode systems. Alternatively, MEG may be used. MEG is the recording of the magnetic fields, generated by the ionic currents at the brain cellular level; thus, both EEG and MEG are methodologically similar and relevant in neuroscience [17]. The second step includes* preprocessing*, eliminating the following artifacts: muscle movements, sleepiness, eye blinks, heartbeat, and other types of EEG “noise.” Preprocessing is performed by selecting “clean” EEG segments for analysis. The last stage is* mathematical processing* of the “clean” (artifact-free) EEG signal to extract a parameter, which denotes best the process of interest (e.g., cognitive decline). Various mathematical approaches are used for the processing; they are generally classified in linear and nonlinear techniques. Linear methods are based on the concept that electric activity of the brain is a stationary process [18]. Nonlinear methods are based on the concept that EEG activity is a dynamic and irregular phenomenon [19]. Each of these methods has its advantages and disadvantages [20, 21].

### 2.2. Spectral Analysis

**Figure 2:**
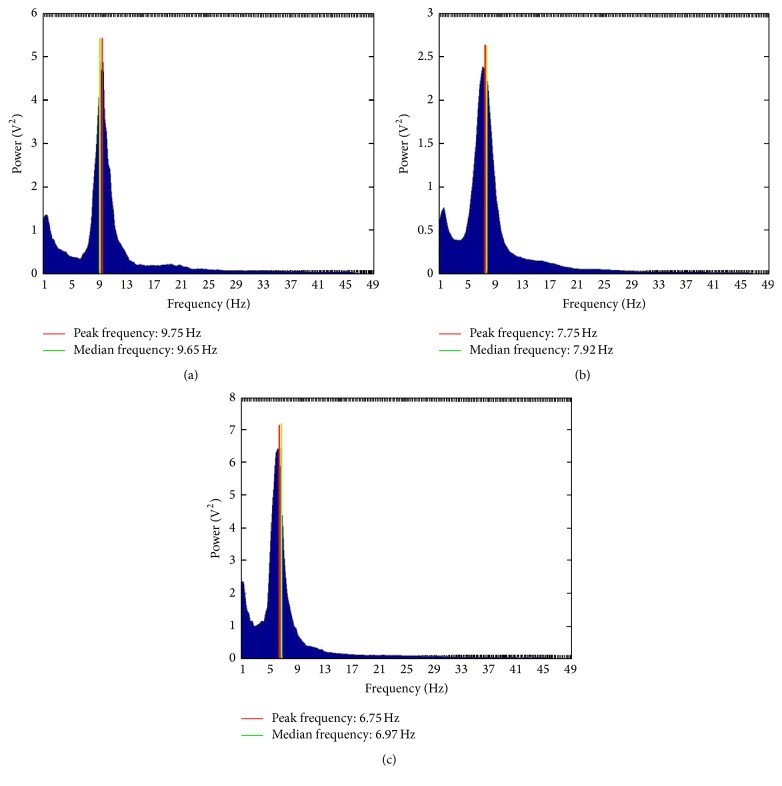
Power spectra of a healthy person (a), a patient with PD-MCI (b), and a patient with PD-D (c); band power: 8–13 Hz. Images computed from our own EEG data using TAPEEG toolbox.

Spectral analysis is a linear technique of EEG processing. It is a process by which a complex EEG signal is decomposed into its component frequencies, and the amplitude of oscillations at each frequency bin is calculated. Since oscillations around zero (like an EEG trace) would add up to 0, amplitudes are represented by their squares, called* power*. The totality of powers at each frequency band is called* power spectrum* and could be represented as a graph (Figure 2). Thus, a power spectrum reflects “the amount of activity” in frequency bands. The frequency bands are the same as those used in conventional EEG, generally consisting of delta (0.1–3.5 Hz), theta (4–7.5 Hz), alpha (8–13 Hz), beta (14–30 Hz), and gamma (>30 Hz) [22]. However, different researchers may select slightly different frequency intervals for their analyses. Additionally, the bands could be divided into subbands, for example, alpha 1 (8–10 Hz) and alpha 2 (10–13 Hz), for the purpose of a thorough analysis.

Spectral power can be absolute or relative.* Absolute power* in a given frequency band, for example, in the alpha band, corresponds to the integral of all power values as measured, while* relative power* is the power in a given frequency band divided by the sum of all power measurements of all frequencies. Additionally, power could be global and regional.* Global power* reflects the average power over the whole cortex, while* regional power* characterizes the power in certain cortex regions. Mainly, 5 regions in each hemisphere are analyzed: frontal, temporal, parietal, occipital, and central, giving a total of 10 regions.

Additionally, some average parameters of EEG frequency can be obtained in spectral analysis [23].* Mean frequency* (also referred to as mean “power frequency” or “spectral center of gravity”) is calculated as the sum of the product of the power spectrum and the frequency divided by the total sum of the power spectrum.* Median frequency* is the 50% quantile of the power spectrum; in other words, it is the frequency at which the power spectrum is divided into two regions with equal amplitude. Finally,* peak frequency* is the frequency which corresponds to the maximum of the power spectrum.

### 2.3. Functional Connectivity Analysis

The other type of information obtained by QEEG (apart from spectral analysis) is functional brain connectivity. Functional connectivity in the context of neuronal activity may be briefly defined as a coordinated interplay between specialized brain regions [24]. Cognitive functions (e.g., attention, memory) arise from neuronal activity, which is distributed over the brain anatomically and temporally, forming complex networks [25]. These networks function on the basis of anatomical connections (white matter tracts connecting brain regions), functional connections (temporal correlations between brain regions, even anatomically unconnected), and effective connections (causal influences between networks) [26]. Thus, functional connectivity analysis is a measure, which enables quantifying the level of the functional connections between brain regions.

As discussed by Bosboom et al. (2009), when performing connectivity analyses, we assume that two dynamically active neural networks are designated “A” and “B” [27]. Time series “*a*_*i*_” and “*b*_*i*_”, using EEG signals from both networks, are recorded. The main purpose is to analyze the functional relation between “A” and “B” from “*a*_*i*_” and “*b*_*i*_” and to quantify the level of this relation. This quantification is performed with both linear and nonlinear methods.

Linear approaches in connectivity analysis assume that the more “*a*_*i*_” and “*b*_*i*_” correspond to each other, the stronger the relation between “A” and “B” is. In this way, for instance, the coherence is calculated as an estimate of a function of frequency between two signals [28]. In contrast to coherence, where the stability of the phase relation between two signals is assessed and taken as an indicator of synchronization between the brain regions, the global field synchronization (GFS) makes no assumption about the spatial location of the activity [29, 30]. GFS is calculated as a function of all frequency bands.

However, there can be a functional relation between the structures “A” and “B” even if time series “*a*_*i*_” and “*b*_*i*_” do not correspond to each other; in this case nonlinear methods of analysis are applied. One of these methods is synchronization analysis, which implies that “*the state of A is a function of the state of B*” [31]. Synchronization likelihood (SL) is an estimate of synchronization, which reflects dynamic interactions of the chaotically active coupled networks. SL denotes how strongly a signal channel at a given time is synchronized to other channels. Another estimate of synchronization is phase lag index (PLI). PLI is calculated from the asymmetry of the distribution of instantaneous signal phase differences between two brain regions and has the advantage of being free of effects of volume conduction as opposed to the methods mentioned before [31]. In other words, PLI reflects the degree of synchronization between couples of signals.

After characterization of single connections, the next level of connectivity analysis consists in description of the whole network, applying graph theory method. In this method functional connections between brain structures are described as graphs (networks) [32]. These graphs consist of vertices (nodes) and corresponding sets of edges (connections). There are different approaches to assess the obtained graph, for example, weighted graph analysis and minimum spanning tree. The two fundamental measures of weighted graph are clustering coefficient (CC) and path length (PL). Olde Dubbelink et al. (2014) describe CC as an estimate of “*the likelihood that neighbors of a vertex are also connected to each other, and characterizes the tendency to form local clusters*” [33]. In other words CC describes local “connectedness.” The same authors described PL as a “*measure for global integration of the network. It is defined as the harmonic mean between all possible vertex pairs in the network, where the shortest path between two vertices is defined as the path with the largest total weight*.” Thus PL describes global “connectedness.”

Graphs may be very complex and large, forming a variety of nodes and paths. A subgraph can be developed which connects all nodes through the shortest paths without forming cycles; such subgraph is referred to as minimum spanning tree of a weighted graph [34]. The following measures are used for minimum spanning tree estimation: leaf number (the number of nodes with only one edge), eccentricity of a node (the length of the longest connection from this node to any other node), betweenness centrality of a node (the fraction of all connections in the tree that include, but do not stop at, that node), and tree hierarchy (a quotient of the leaf number to the product of twice the number of edges to the highest betweenness centrality of any node in the tree). These measures estimate the complexity of connections in the topographical brain network [34]. There are other various types of connectivity analysis, but we briefly described only those, which will be referred to further in the text of this review.

## 3. Reliability of the QEEG Analysis

### 3.1. Individual Variability

According to Näpflin et al. (2007) interindividual variability of absolute power of the traditional frequency bands in healthy humans is large, while intraindividually the power spectrum remains stable over a period of 12 to 40 months in healthy individuals [61].

However, interpretation of a change in relative power in an individual is ambiguous and requires knowledge of more information than a change in absolute power. For example, a decrease of the relative alpha power can be due to either a decrease of absolute alpha power but also to an increase of the absolute power in one or more of the other frequency bands without any change in the absolute alpha power or to a combination of both. In cross-sectional comparisons of small groups of individuals, alterations in relative power are more easily detected than changes in absolute power, while absolute power is a good measure for longitudinal, intraindividual changes or cross-sectional comparisons of very large populations. Derived indices were proposed as a possible solution for the problem that exists in relative power relationship between frequency bands: spectral ratio (sum of alpha and beta powers divided by the sum of delta and theta powers) [40] or alpha/theta ratio [43].

### 3.2. Test-Retest Effect

According to consecutive reports EEG frequency parameters are stable over time. Gasser et al. (1985) were amongst the first to address the issue of test-retest reliability of EEG parameters [62]. They reported that alpha electrical activity of the brain cortex showed the best reliability and delta and beta activity had the worst reliability. Dustman et al. (1999) investigated the variability of absolute and relative powers in five frequency bands, delta, theta, alpha, beta, and gamma, over the interval of 6 months in a sample of 222 males aged from 4 to 90 years [63]. Age-related dependence of the parameters was identified, but the frequency markers, especially power in the alpha band, showed a satisfactory reliability over time. Later, Näpflin et al. (2007), in the above-mentioned study, replicated these results in healthy adults [61].

Additionally, the EEG frequency markers are not influenced by cognitive activity. Grandy et al. (2013) investigated the modifiability of the alpha frequency of healthy subjects before and after a series sessions of cognitive tasks [64]. Cognitive tasks had no significant effects on the resting state peak alpha frequency 7.5–12.5 Hz.

### 3.3. Influence of Dopamine-Replacement Therapy on QEEG Parameters

The effects of levodopa and dopaminergic medication on the EEG activity of the patients yielded ambiguous results: while some researchers reported that patients in a medicated and a nonmedicated state revealed no influence of dopamine-replacement therapy on frequency characteristics [49, 65], various other studies reported that levodopa treatment of PD induces an increase in alpha and beta bands and a decrease of theta and delta bands. These latter changes are referred to as “activation” of EEG [66].

George et al. (2013) analyzed the EEG power spectra and connectivity in nondemented PD patients in ON- and OFF-medication state, in both resting state and during a cognitive task [67]. These results were compared to those of a group of healthy controls. No significant changes in powers were identified in relation to medication. Despite that fact, the authors showed that dopaminergic medication reduced the pathological synchronization in the beta band in the resting state and induced task-related increase of beta power. These findings were consistent with the previous reports [50, 68]. According to other researchers levodopa treatment has influence on functional brain connectivity assessed by MEG and these changes were mostly identified in beta frequency range [69]. Therefore, studies of beta activity require adjustments according to dopaminergic stimulation while data with alpha and theta activity is probably largely independent from dopaminergic influence.

## 4. Spectral Characteristics of Cognitive States in PD

### 4.1. Global Power Spectra

Seventeen studies focused on spectral features of cognitive states in PD. Six of these 17 studies focused on the capacity of discrimination between better and worse states of cognition in PD (e.g., group of patients with PD-MCI versus group with PD patients with normal cognition (PD-NC) or group with PD-MCI versus group with PD-D) [35, 36, 42, 43, 46, 48] (Table 2). Global delta and theta powers (these variables were increased in PD-D patients) and peak background frequency (decreased in PD-D patients) had the largest effect sizes to discriminate PD-NC versus PD-D. Global delta power (increased in PD-D patients), peak background frequency, and global alpha power (decreased in PD-D patients) had the largest effect sizes to distinguish PD-MCI versus PD-D. Additionally, beta peak frequency was significantly increased (*p* < 0.01), and global alpha power and alpha/theta ratio were significantly decreased (*p* < 0.01 and *p* < 0.01) in PD-D versus PD-MCI in one report (although original data was not available) [43]. Global alpha power, peak background frequency (decreased in PD-MCI patients), and global theta power (increased in PD-MCI patients) had the largest effect sizes to discriminate PD-NC versus PD-MCI.

Patients with PD-D were compared to PD patients without dementia in two studies [42, 48]. The latter group might include both PD-NC and PD-MCI. However, global delta and theta powers (increased in PD-D patients) had the largest effect sizes. In one study, two groups of patients with PD-D, with cognitive fluctuations (CF) and without CF, were compared by the analysis of the compressed spectral arrays (CSA) [36]. CF are described as disorders of consciousness ranging from reduced arousal to stupor; CF indicate a worse state of dementia [56]. CSA is a method of epoch-to-epoch QEEG representation for each derivation, CSA provide information on various QEEG parameters like spectral powers, dominant frequency (DF), mean frequency where the maximal power is represented in the sum of all epochs, DF variability (DFV) across all analyzed epochs, and other parameters. Global alpha and prealpha (5.6–7.9 Hz) powers had the largest effect sizes: alpha was decreased and “prealpha” was increased in patients with PD-D and CF.

### 4.2. Topographic Distribution of Power Spectra

Topographic distribution of spectral powers was addressed in 7 studies [36–38, 40, 46, 48, 51]. Theta and alpha powers in temporal and parietal regions bilaterally had the largest effect sizes to distinguish between PD-NC and PD-D patients. Theta power was increased and alpha power decreased in PD-D patients. Spectral ratio (sum of alpha and beta powers divided by the sum of delta and theta powers) in frontal regions and delta and alpha powers in posterior derivations had the largest effect sizes to distinguish between PD-MCI and PD-D. Delta power was increased and alpha power and spectral ratio were decreased in PD-D patients. Theta and beta powers and spectral ratio in posterior derivations had the largest effect sizes to distinguish between PD-NC and PD-MCI. Theta power was increased and alpha power was decreased in PD-MCI patients. In one study PD patients with executive dysfunction were compared to PD patients without executive dysfunction [38]. The largest effect size had spectral ratio in frontal derivations; spectral ratio was decreased in patients with executive dysfunction. Additionally, in one study PD-D patients were compared with PD without dementia [48]. The largest effect sizes had alpha and delta powers in temporal, parietal, and occipital regions and beta and delta powers in central regions, and beta, alpha, and delta powers in frontal regions. Delta power was increased, and alpha and beta powers were decreased in PD-D patients. Finally, prealpha, DF, and DFV in frontal, temporal, and parietooccipital derivations had the largest effect size for distinguishing PD-D patients without CF from PD-D patients with CF [36]. Prealpha and DFV were increased and DF was decreased in patients with PD-D and CF.

### 4.3. Correlation of Power Spectra with Cognitive Assessment Tools

Correlation of spectral powers with different cognitive assessment tools and tests was analyzed in 7 studies [35, 39, 40, 45, 47, 48, 50]. The details are presented in Table 3. The mostly used tool for cognitive assessment in these studies was the MMSE. Positive Fisher's *Z* was observed for Mini-Mental State Examination (MMSE) and spectral ratios at all scalp locations, relative power in the range 8–13 Hz (alpha), and peak background frequency, while negative Fisher's *Z* was observed for MMSE and relative power in the range 0–4 Hz (delta). Negative Fisher's *Z* was observed for Cambridge Cognitive Examination (CAMCOG) and relative power in the range 4–8 Hz (theta) in bilateral occipital and right temporal regions. Additionally, in one study, correlation of median frequency with cognitive domains was investigated [47]. Significant correlations were observed for “episodic and long term memory domain,” followed by “overall cognitive score,” “fluency domain,” “attention domain,” and “executive functions domain.” In one study no correlation of absolute power spectra with neuropsychiatric inventory was reported in nondemented PD patients [45].

Additionally, longitudinal correlation of frequency results with cognitive states in PD using tools for cognitive assessment was assessed in 3 studies [36, 44, 52]. In the first study [36], correlation with Frontal Assessment Battery scores was investigated: negative Fisher's *Z* was observed for power in the range 8–12 Hz (alpha) and positive Fisher's *Z* for powers in the range 4–8 Hz (theta), over 2 years [36]. In another study [52], various tools for cognitive assessment correlated with power spectra over 7 years of observation: negative Fisher's *Z* was observed: for global relative powers (GRP) in the range 0.5–4 Hz (delta) and CAMCOG and Spatial Span Test (SSP); for GRP in the range 4–8 Hz (theta) and CAMCOG, Pattern Recognition Memory (PRM), Semantic Fluency Test, and Spatial Span Test; for GRP in the range 8–10 Hz (alpha 1) and Spatial Working Memory (SWM). Positive Fisher's *Z* was observed: for powers in the range 8–13 Hz (alpha 1 and alpha 2) and 30–48 Hz (gamma) and CAMCOG, PRM, and SSP; for powers in the range 4–8 Hz (theta) and SWM [45]. In the third study [44], correlation with power in the range 2.5–4 Hz (delta) was investigated: negative Fisher's *Z* was observed for MMSE, Rey Auditory Verbal Learning, Controlled Oral Word Association Test and Stroop, while positive Fisher's *Z* was observed for Clinical Dementia Rating Sum of Boxes and Functional Assessment Staging Tool.

### 4.4. Hazard of Conversion to PD-D

The relation of power spectra to conversion to PD-D was examined in 3 studies [9, 43, 54]. The details are presented in Table 4. Hazard ratios of conversion to PD-D were analyzed in 2 studies. The hazard ratio of conversion to PD-D was significantly higher for patients with background EEG frequency below the median value of the entire sample at baseline [9] and the theta power above the median value of the entire sample at baseline [54]. In one study, patients with PD-MCI who converted to PD-D over two years had increased beta peak frequency and decreased alpha relative power and alpha/theta ratio at baseline [43].

## 5. Brain Functional Connectivity and Cognitive States in PD

Seven studies focused on functional connectivity features of cognitive states in PD [27, 33, 41, 42, 50–52]. Global field synchronization (GBS) was addressed in one study and coherence in another one. Patients with PD-D were compared with PD patients without dementia in both studies. PD-D patients had significantly higher GBS in theta frequency range (*p* < 0.02) and lower GBS in the alpha 1 range (*p* < 0.02) [41]; higher frontal interhemispheric (F3-F4) and higher frontooccipital intrahemispheric (F3-O1; F4-O2) coherence in the beta frequency band was observed in another study [42].

In two studies SL was investigated. In one study correlation of connectivity markers with cognitive tests in PD patients without dementia and with varying disease duration was investigated [50]. Higher level of perseveration executive task in patients with recently diagnosed PD (in the last 6 months before participation in the study) was associated with increased interhemispheric SL in alpha 1 band. In an exploratory study by Bosboom et al. (2009) PD-D patients were compared to nondemented PD patients [27]. Patients with PD-D had lower interhemispheric SL between temporal regions (frequency ranges: 0.5–4 Hz, 4–8 Hz and 8–10 Hz) and parietal regions (30–48 Hz); lower intrahemispheric SL between frontal and temporal and frontal and parietal regions in the left hemisphere (8–13 Hz) and frontal and temporal regions in the right hemisphere (8–13 Hz and 13–30 Hz). At the same time, higher intrahemispheric SL was found between occipital and temporal and occipital and parietal regions in the left hemisphere (13–30 Hz) and between parietal and occipital regions in the right hemisphere (8–10 Hz).

Phase lag index (PLI) was investigated in two studies. A comparison of PD-D patients with nondemented PD patients showed weaker PLI in frontotemporal (0.5–4 Hz) and parietotemporooccipital (8–13 Hz) couplings in demented patients [51]. In this study, general region-to-region connectivity was stronger in theta band and weaker in delta, alpha, and beta bands in PD-D. A longitudinal observation of initially nondemented PD patients showed correlation of worsening of CAMCOG performance with a decrease of PLI in frontal and temporal regions in frequency range 8–10 Hz [53]. Finally, a graph theory analysis of longitudinal connectivity changes of nondemented PD patients was performed in one study [33]. Worsening of cognitive performance over time correlated with increase in eccentricity in the frequency range 8–10 Hz and decrease of clustering coefficient and path length in the frequency range 4–8 Hz.

## 6. Conclusions

The results of this review support the idea that spectral and connectivity markers have a significant impact in discriminating PD patients with different levels of cognitive decline, regardless of the variety of approaches to calculate these markers. To summarize, a slowing of EEG frequencies correlates with a decline of cognition. Accordingly, an increase of spectral powers in the “slow” frequency bands <8 Hz (delta and theta) and a decrease in the “fast” frequency bands >8 Hz (alpha, beta, and, less significantly, gamma) are spectral markers of PD-related cognitive decline. Topographically, occipital, parietal, and temporal regions show the higher significance.

Additionally, the above-mentioned spectral markers showed significant hazard ratio in predicting conversion of nondemented PD patients to PD-D. Patients with spectral powers in “fast” waves below and in “slow” waves above the median values have significantly higher risk of developing PD-D within 2 to 7 years.

The connectivity patterns of the PD patients with cognitive impairment show changes in the same frequency ranges, where spectral markers of cognitive decline are identified: mostly in theta (4–8 Hz), alpha 1 (8–10 Hz), and beta (13–30 Hz) ranges. The connectivity patterns of PD patients with cognitive decline changed in frontal, temporal, parietal, and occipital regions. However, the number of connectivity studies focusing on cognitive states of PD patients is still very small; by the same token the studies had different setting and various connectivity markers were investigated. A common trend of cognitive decline in PD seems to be a decrease of connectivity in parietotemporooccipital regions.

In sum, changes in spectral powers, delta and theta, have the highest significance to discriminate between PD-D and dementia-free patients with PD, while changes in spectral powers, theta and alpha, have the highest significance to separate MCI from normal cognition in PD. Findings regarding discrimination between MCI and dementia in PD are less consistent within reports, though delta and beta powers showed good discriminative capacity. With regard to connectivity measures, PLI has the highest significance to discriminate between PD-D and nondemented patients with PD.

Importantly, changes of spectral QEEG markers precede the clinical manifestation of cognitive decline in PD, as was shown in longitudinal studies. Thus, these markers may become a valuable aid for timely selection of patients prone to pharmacological and nonpharmacological interventions of prevention at a very early stage of PD and thereby potentially improve clinical results.

Prospective studies with larger cohorts investigating topographical scalp distribution of QEEG changes as well as connectivity and its association with cognitive decline in PD are warranted. These studies will result in biomarkers that are likely to contribute to individualized counselling and treatment of patients.

## 7. Limitations of This Review

This review has several limitations. First, there is no common opinion regarding which certain markers can be used to predict cognitive decline in PD. By virtue of various fast developing methods and approaches, different research groups investigate different methods: spectral markers, connectivity markers, or their combination. In these conditions a thorough comparison of QEEG markers remains a challenge. However, future methods might further improve the validity of QEEG biomarkers of cognitive decline in PD.

Second, criteria for the diagnosis of PD-MCI are changing over time [12, 55]. In some studies a simple cognitive screening is performed using Mini-Mental State Examination tool; in other cases a full cognitive assessment is performed with many cognitive tests. Since 2012 the Movement Disorders Society Task Force guidelines set a common criteria for PD-MCI [58]; however, the Diagnostic and Statistical Manual of Mental Disorders fifth edition has replaced the term MCI by “neurocognitive impairment” in 2013 [70].

In sum, while differentiation between patients with PD with an intact cognitive state and patients with PD-D could be performed more or less clearly using QEEG markers, identification of the borderline level of cognition is relatively difficult.

## Supplementary Material

Supplementary Material displays the flow chart of the review process (1) and the list of the publications which were excluded after the analysis of the full text with reasons of exclusion explained (2).

## Figures and Tables

**Table 1:** Profiles of the studies, which met the inclusion criteria.

Number	Author(s)	Type of the study/setting	Analyzed parameter(s)	Affiliation of the corresponding author
Studies with EEG with 10-20 international system				
1	Caviness et al. 2007 [35]	Comparison of 8 PD-D patients versus 16 PD-MCI patients versus 42 PD-NC patients	Relative spectral power	Mayo Clinic, Scottsdale, USA
2	Bonanni et al. 2008 [36]	Observation of 36 LBD patients, 19 PD-D patients without cognitive fluctuations, 16 PD-D patients with cognitive fluctuations, 17 AD patients, and 50 HC	Compressed spectral arrays and relative spectral power	G. d'Annunzio University of Chieti-Pescara, Pescara, Italy
3	Fonseca et al. 2009 [37]	Comparison of 7 PD-D patients versus 10 PD-MCI patients versus 15 PD-NC patients versus 26 HC	Relative and absolute amplitudes	Pontificia Universidade Catolica de Campinas, Campinas, Brazil
4	Kamei et al. 2010 [38]	Comparison of PD patients with executive dysfunction versus 25 PD patients without executive dysfunction	Absolute spectral power	Nihon University School of Medicine, Tokyo, Japan
5	Babiloni et al. 2011 [39]	Comparison of 13 PD-D patients versus 20 AD patients versus 20 HC	Spectral and source analyses	Casa di Cura San Raffaele Cassino, Italy
6	Klassen et al. 2011 [9]	Observation of 106 PD-wD patients	Relative spectral power	Mayo Clinic, Scottsdale, USA
7	Morita et al. 2011 [40]	Comparison of 100 PD patients: 43 with MMSE 28–30 versus 35 with MMSE 24–27 versus 22 with MMSE <24	Absolute spectral power	Nihon University School of Medicine, Tokyo, Japan
8	Pugnetti et al. 2010 [41]	Comparison of 21 PD-wD patients versus 7 PD-D patients versus 10 LBD patients versus 14 HC	Global field synchronization	Scientific Institute of S. Maria Nascente, Milan, Italy
9	Fonseca et al. 2013 [42]	Comparison of 12 PD-D patients versus 31 PD-wD patients versus 38 AD patients versus 37 HC	Absolute spectral power and coherence	Pontificia Universidade Catolica de Campinas, Campinas, Brazil
10	Gu et al. 2016 [43]	Observation of 9 PD-D patients and 17 PD-MCI patients	Relative and absolute spectral power	Nanfang Hospital, Guangzhou, China
11	Caviness et al. 2015 [44]	Observation of 71 PD-wD patients	Relative spectral power	Mayo Clinic, Scottsdale, USA
12	Fonseca et al. 2015 [45]	Comparison of 31 PD-wD patients versus 28 AD patients versus 27 HC	Absolute spectral power and coherence	Pontificia Universidade Catolica de Campinas, Campinas, Brazil

Studies with EEG with 256 channels				
13	Bousleiman et al. 2014 [46]	Comparison of 12 PD-NC patients versus 41 PD-MCI patients	Relative spectral power	Hospital of the University of Basel, Basel, Switzerland
14	Zimmermann et al. 2014 [47]	Analysis of 48 PD-wD patients	Median background frequency	Hospital of the University of Basel, Basel, Switzerland

Studies with 151-channel whole-head MEG				
15	Bosboom et al. 2006 [48]	Comparison of 13 PD-D patients versus 13 PD-wD patients versus 13 HC	Relative spectral power	VU University Medical Center, Amsterdam, the Netherlands
16	Stoffers et al. 2007 [49]	Comparison of 70 PD-wD patients versus 21 HC	Relative spectral power	VU University Medical Center, Amsterdam, the Netherlands
17	Stoffers et al. 2008 [50]	Comparison of 70 PD-wD patients versus 21 HC	Synchronization likelihood	VU University Medical Center, Amsterdam, the Netherlands
18	Bosboom et al. 2009 [27]	Comparison of 13 PD-D patients versus 13 PD-wD patients	Synchronization likelihood	VU University Medical Center, Amsterdam, the Netherlands
19	Ponsen et al. 2013 [51]	Comparison of 13 PD-D patients versus 13 PD-wD patients	Relative spectral power and phase lag index	VU University Medical Center, Amsterdam, the Netherlands
20	Olde Dubbelink et al. 2013 [52]	Observation of 49 PD-wD patients and 14 HC	Relative spectral power	VU University Medical Center, Amsterdam, the Netherlands
21	Olde Dubbelink et al. 2013 [53]	Observation of 43 PD-wD patients and 14 HC	Phase lag index	VU University Medical Center, Amsterdam, the Netherlands
22	Olde Dubbelink et al. 2014 [33]	Observation of 43 PD-wD patients and 14 HC	Weighted graph and minimum spanning tree	VU University Medical Center, Amsterdam, the Netherlands
23	Olde Dubbelink et al. 2014 [54]	Observation; 63 PD-wD patients	Relative spectral power	VU University Medical Center, Amsterdam, the Netherlands

AD: Alzheimer's disease; DLB: dementia with Lewy bodies; HC: healthy controls; PD-D: Parkinson's disease with dementia; PD-MCI: Parkinson's disease with mild cognitive impairment; PD-NC: Parkinson's disease with normal cognition; PD-wD: Parkinson's disease without dementia.

**Table 2:** EEG and MEG spectral markers which significantly discriminated between cognitive states in PD.

Author(s)	Diagnostic groups of patients with PD (*N*)	Mean age (years)	Evaluative tests: cognitive pathology (criteria)	Parameter(s) showed significant difference between the groups with PD	Effect size (95% CI)
Bosboom et al. 2006^a^ [48]	PD-D (13) PD-wD (13)	74.4 71.7	Dementia (DSM-IV)	GRP delta (0.5–4 Hz) and GRP theta (4–8 Hz)	*PD-wD versus PD-D* 1.47 (0.60, 2.34)
				GRP alpha (8–13 Hz) and GRP beta (13–30 Hz)	*PD-wD versus PD-D* −1.47 (−2.34, −0.60)
				GRP gamma (30–48 Hz)	*PD-wD versus PD-D* −1.47 (−2.34, −0.60)

Caviness et al. 2007 [35]	PD-D (8) PD-MCI (16) PD-NC (42)	78.0 80.4 74.6	Dementia (DSM-IV); MCI (Petersen 2004 [55])	GRP delta (1.5–3.9 Hz)	*PD-NC versus PD-MCI* 0.11 (−0.47, 0.68) *PD-MCI versus PD-D* 1.27 (0.35, 2.19) *PD-NC versus PD-D* 1.46 (0.67, 2.29)
				GRP theta (4–7.9 Hz)	*PD-NC versus PD-MCI* 0.75 (0.16, 1.34) *PD-MCI versus PD-D* 0.38 (−0.46, 1.24) *PD-NC versus PD-D* 1.37 (0.57, 2.17)
				GRP alpha (8–12.9 Hz)	*PD-MCI versus PD-D* −0.86 (−1.75, 0.01) *PD-NC versus PD-D* −1.01 (−1.79, −0.22)
				GRP beta 1 (13–19.9 Hz)	*PD-NC versus PD-MCI* −0.63 (−1.21, 0.04) *PD-MCI versus PD-D* −0.70 (−1.57, 0.17) *PD-NC versus PD-D* −1.16 (−1.95, −0.37)
				GRP beta 2 (20–30 Hz).	*PD-NC versus PD-MCI* −0.57 (−1.15, 0.02) *PD-MCI versus PD-D* −0.81 (−1.69, 0.07) *PD-NC versus PD-D* −1.21 (−2.00, −0.41)
				Peak frequency at locations P3, P4, and Oz	*PD-NC versus PD-MCI* −0.90 (−1.51, −0.31) *PD-MCI versus PD-D* −0.99 (−1.88, −0.10) *PD-NC versus PD-D* −1.88 (−2.54, −1.20)

Bonanni et al. 2008^b^ [36]	PD-DnF (19) PD-DF (16)	70.0^c^	PD-D (history of PD preceded dementia for at least 24 months); cognitive fluctuations (CAF, Walker et al. 2000 [56])	GRP theta (4.0–5.5 Hz)	*PD-DnF versus PD-DF* 2.82 (1.88, 3.75)
				GRP prealpha (5.6–7.9 Hz)	*PD-DnF versus PD-DF* 5.26 (3.86, 6.67)
				GRP alpha (8.0–12.0 Hz)	*PD-DnF versus PD-DF* −8.40 (−10.47, −6.32)
				Mean frequency	*PD-DnF versus PD-DF* −0.93 (−1.64, −0.24)
				DF in parietooccipital derivations	*PD-DnF versus PD-DF* −1.18 (−1.90, −0.46)
				DFV in parietooccipital derivations	*PD-DnF versus PD-DF* 1.19 (0.47, 1.91)

Fonseca et al. 2013 [42]	PD-D (12) PD-wD (31)	70.3 68.1	Dementia (Dubois et al. 2007 [57])	Mean absolute power delta (0.8–3.9 Hz)	*PD-wD versus PD-D* 0.85 (0.16, 1.54)
				Mean absolute power theta (4.29–7.8 Hz)	*PD-wD versus PD-D* 1.23 (0.52, 1.94)

Bousleiman et al. 2014 [46]	PD-MCI (41) PD-NC (12)	67.2^c^	MCI (Litvan et al. 2012 [58]).	GRP alpha 1 (8–10 Hz)	*PD-NC versus PD-MCI* −0.82 (−0.131, −0.001)

Gu et al. 2016^a,b^ [43]	PD-D (9) PD-MCI (17)	56.7^d^ 62.1^d^	Dementia (DSM-IV); MCI (Petersen 2004 [55])	Beta (13–30 Hz) peak frequency	*PD-MCI versus PD-D* 1.10 (0.27, 1.92)
				GRP alpha (8–13 Hz)	*PD-MCI versus PD-D* −1.10 (−1.92, −0.27)
				alpha/theta ratio: alpha (8–13 Hz) divided by theta (4–7 Hz)	*PD-MCI versus PD-D* −1.10 (−1.92, −0.27)

^a^Original data not available, effect size and confidence intervals estimated using *p* value conversion.

^b^The study is longitudinal; only assessment on admission is shown in this table.

^c^Age for groups of the patients is not available; age of the combined sample is shown.

^d^Mean age not available, mean age calculated from median and range (Hozo et al. 2005 [59]).

CAF: Clinical Assessment of Fluctuations; DF: dominant frequency; DFV: dominant frequency variability; DSM-IV: Diagnostic and Statistical Manual of Mental Disorders IV; GRP: global relative power; MCI: mild cognitive impairment; PD: Parkinson's disease; PD-NC: Parkinson's disease without cognitive impairment; PD-MCI: Parkinson's disease with mild cognitive impairment; PD-D: Parkinson's disease with dementia; PD-wD: Parkinson's disease without dementia; PD-DnF: Parkinson's disease with dementia without cognitive fluctuations; PD-DF: Parkinson's disease with dementia with cognitive fluctuations.

**Table 3:** Markers which significantly correlated with various cognitive assessment tools in PD.

Author(s)	Age, mean	*N*	Correlation	Fisher's *Z* (95% CI)
Bosboom et al. 2006 [48]	71.7	13 PD-wD patients	Left occipital theta (4–8 Hz) versus CAMCOG	−0.70 (−1.32, 0.08)
			Right occipital theta (4–8 Hz) versus CAMCOG	−0.67 (−1.29, 0.05)
			Right temporal theta (4–8 Hz)	−0.68 (−1.30, 0.06)

Caviness et al. 2007 [35]	76.4	66 PD-wD patients	GRP delta (1.5–3.9 Hz) versus MMSE	−0.51 (−0.76, −0.26)
			GRP alpha (8–12.9 Hz) versus MMSE	0.34 (0.10, 0.59)
			Peak background frequency versus MMSE	0.42 (0.18, 0.67)

Stoffers et al. 2008 [50]	59.4	18 *de novo* PD patients	Relative low alpha (8–10 Hz) versus redundancy of the second order (Vienna perseveration) in bilateral central and parietal regions	−0.11 (−0.19, −0.01)

Morita et al. 2011 [40]	67.6	100 PD patients	Spectral ratio (SR^a^) at Fp location (electrode positions Fp1 and Fp2) versus MMSE	0.30 (0.10, 0.50)
			SR at F location (electrode positions F3, F4, F7, and F8) versus MMSE	0.32 (0.12, 0.52)
			SR at C location (electrode positions C3 and C4) versus MMSE	0.28 (0.08, 0.48)
			SR at P location (electrode positions P3 and P4) versus MMSE	0.32 (0.12, 0.52)
			SR at T location (electrode positions T3, T4, T5, and T6) versus MMSE	0.32 (0.12, 0.52)
			SR at O location (electrode positions O1 and O2) versus MMSE	0.35 (0.16, 0.55)

Babiloni et al. 2011 [39]	72.0	13 PD-D patients	Relative alpha1 (8–10.5 Hz) in parietal regions (Brodmann areas 5, 7, 30, 39, 40, and 43) versus MMSE	0.35 (−0.27, 0.97)
			Relative alpha1 (8–10.5 Hz) in occipital regions (Brodmann areas 5, 7, 30, 39, 40, and 43) versus MMSE	0.44 (−0.18, 1.05)

Fonseca et al. 2015 [45]	68.8	31 PD-wD patients	Absolute powers: delta (0.8–3.9 Hz), theta (4.29–7.8 Hz), alpha (8.2–12.5 Hz), and beta (12.9–36.3 Hz) versus neuropsychiatric inventory	No significant correlation with any marker

Zimmermann et al. 2014 [47]	67.6	48 PD-wD patients	Median frequency versus episodic and long term memory cognitive domain (CD^b^)	0.60 (0.31, 0.90)
			Median frequency versus overall cognitive score^c^	0.51 (0.22, 0.80)
			Median frequency versus fluency CD	0.41 (0.12, 0.70)
			Median frequency versus attention CD	0.39 (0.10, 0.68)
			Median frequency versus executive functions CD	0.35 (0.06, 0.65)

Original data not available in the publications. Fisher's *Z* calculated from correlation coefficient and sample size (Lipsey and Wilson, 2001 [60]).

^a^Sum of absolute power values for alpha (8.20–12.89 Hz) and beta (13.28–30.8 Hz); waves divided by the sum of absolute power values for delta (1.17–3.91 Hz) and theta (4.3–7.81 Hz).

^b^Parameter, which includes a set of cognitive tests from a specific cognitive category, for example. memory and attention.

^c^Parameter, which includes an average of 26 cognitive tests from all cognitive domains.

CAMCOG: Cambridge Cognition Examination; GRP: global relative power; MMSE: Mini-Mental State Examination; PD-D: Parkinson's disease with dementia; PD-wD: Parkinson's disease without dementia.

**Table 4:** Prediction of progression to dementia in Parkinson's disease with spectral EEG markers.

Author(s)	Number of subjects, duration of observation after baseline EEG/MEG	Incidence of PD-D	Significant QEEG risk factor(s)
Klassen et al. 2011 [9]	*N* = 106 PD-wD patients, 0.3 to 8.8 (mean 3.3) years	Incidence within 5 years by Kaplan-Meier method was 34%	Hazard ratios: background rhythm frequency < median (8.5) was 13.0; theta power > median (19.0) was 3.0

Gu et al. 2016 [43]	*N* = 17 PD-MCI and 9 PD-D patients, 2 years	35% (6 PD-MCI patients progressed to PD-D patients)	Increase of the beta peak frequency and decrease of alpha relative power and alpha/theta ratio correlated with progression to PD-D; PPV of the combined marker was 62, and PLR was 4.4

Olde Dubbelink et al. 2014 [54]	*N* = 63 PD-wD patients, 7 years	30% (19 patients)	Hazard ratios: beta power < median (27.96) was 5.21; peak frequency < median (8.39) was 3.97; theta power > median (22.85) was 2.82

PD-D: Parkinson's disease with dementia; PD-MCI: Parkinson's disease with mild cognitive impairment; PD-wD: Parkinson's disease without dementia; PPV: positive predictive value; PLR: positive likelihood ratio.
