# Supplementary material for: Quantitative EEG and Cognitive Decline in Parkinson's Disease
Source: Parkinsons Dis. 2016 Apr 11;2016:9060649. doi: 10.1155/2016/9060649 (PMC4842380; doi:10.1155/2016/9060649)
Supplement: Supplementary file 1 — Supplementary Material displays the flow chart of the review process (1) and the list of the publications which were excluded after the analysis of the full text with reasons of exclusion explained (2). [file 9060649.f1.pdf]

Supplement 1. Flow chart of the review process

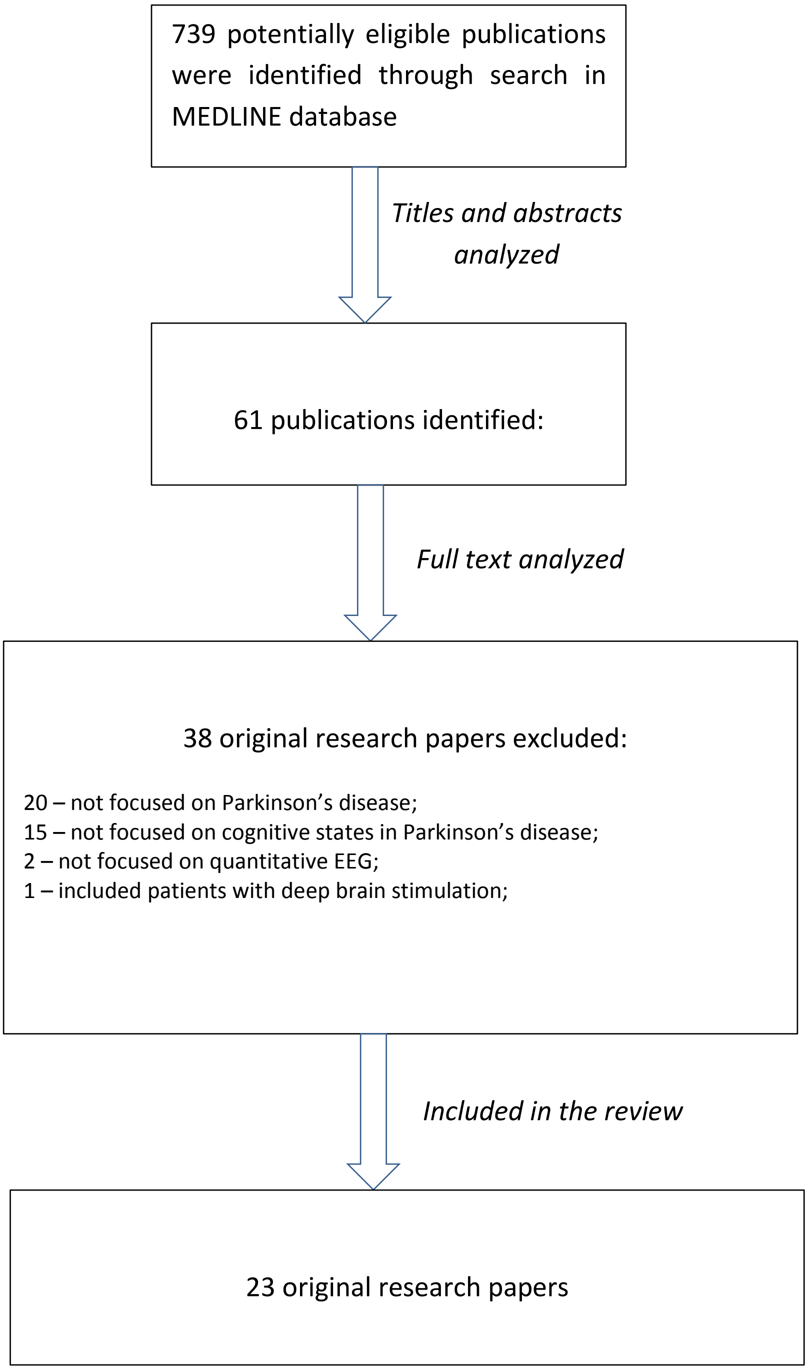

Supplement 2. List of the excluded publications

No	Reference	Cause of exclusion
1.	Kai T, Asai Y, Sakuma K, Koeda T, Nakashima K. Quantitative electroencephalogram analysis in dementia with Lewy bodies and Alzheimer's disease. J Neurol Sci. 2005;237(1-2):89-95.	Not focused on PD.
2.	Koenig T, Prichep L, Dierks T, Hubl D, Wahlund LO, John ER, Jelic V. Decreased EEG synchronization in Alzheimer's disease and mild cognitive impairment. Neurobiol Aging. 2005;26(2):165-171.	Not focused on PD.
3.	Babiloni C, Binetti G, Cassetta E, Dal Forno G, Del Percio C, Ferreri F, Ferri R, Frisoni G, Hirata K, Lanuzza B, Miniussi C, Moretti DV, Nobili F, Rodriguez G, Romani GL, Salinari S, Rossini PM. Sources of cortical rhythms change as a function of cognitive impairment in pathological aging: a multicenter study. Clin Neurophysiol. 2006;117(2):252-268.	Not focused on PD.
4.	Babiloni C, Frisoni G, Steriade M, Bresciani L, Binetti G, Del Percio C, Geroldi C, Miniussi C, Nobili F, Rodriguez G, Zappasodi F, Carfagna T, Rossini PM. Frontal white matter volume and delta EEG sources negatively correlate in awake subjects with mild cognitive impairment and Alzheimer's disease. Clin Neurophysiol. 2006;117(5):1113-1129.	Not focused on PD.
5.	Prichep LS, John ER, Ferris SH, Rausch L, Fang Z, Cancro R, Torossian C, Reisberg B. Prediction of longitudinal cognitive decline in normal elderly with subjective complaints using electrophysiological imaging. Neurobiol Aging. 2006;27(3):471-481.	Not focused on PD.
6.	Che H, Jung YJ, Im CH, Lee S. Extraction of qEEG variables to diagnose early dementia. Conf Proc IEEE Eng Med Biol Soc. 2007;2007:4115-4118.	Not focused on PD.
7.	Gawel M, Zalewska E, Szmidt-Sałkowska E, Kowalski J. Does EEG (visual and quantitative) reflect mental impairment in subcortical vascular dementia? J Neurol Sci. 2007;257(1-2):11-16.	Not focused on PD.
8.	Andersson M., Hansson O., Minthon L., Rosén I., Londos E. Electroencephalogram variability in dementia with Lewy bodies, Alzheimer's disease and controls. Dement. Geriatr. Cogn. Disord. 2008;26:284–290.	Not focused on PD.
9.	Luckhaus C, Grass-Kapanke B, Blaeser I, Ihl R, Supprian T, Winterer G, Zielasek J, Brinkmeyer J. Quantitative EEG in progressing vs stable mild cognitive impairment (MCI): results of a 1-year follow-up study. Int J Geriatr Psychiatry. 2008;23(11):1148-1155.	Not focused on PD.
10.	Moazami-Goudarzi M, Sarnthein J, Michels L, Moukhtieva R, Jeanmonod D. Enhanced frontal low and high frequency power and synchronization in the resting EEG of parkinsonian patients. Neuroimage. 2008;41(3):985-997.	Not focused on cognitive states.
11.	Onishi J, Suzuki Y, Yoshiko K, Hibino S, and Iguchi A. Predictive Model for Assessing Cognitive Impairment by Quantitative Electroencephalography. Cog Behav Neurol 2005;18(3):179-184.	Not focused on PD.
12.	Roks G, Korf ES, van der Flier WM, Scheltens P, Stam CJ. The use of EEG in the diagnosis of dementia with Lewy bodies. J Neurol Neurosurg Psychiatry. 2008;79(4):377-380.	Not focused on PD.
13.	Rossini PM, Buscema M, Capriotti M, Grossi E, Rodriguez G, Del Percio C, Babiloni C. Is it possible to automatically distinguish resting EEG data of normal elderly vs. mild cognitive impairment subjects with high degree of accuracy? Clin Neurophysiol. 2008;119(7):1534-1545	Not focused on PD.
14.	Serizawa K, Kamei S, Morita A, Hara M, Mizutani T, Yoshihashi H, Yamaguchi M, Takeshita J, Hirayanagi K. Comparison of quantitative EEGs between Parkinson disease and age-adjusted normal controls. J Clin Neurophysiol. 2008;25(6):361-366.	Not focused on cognitive states.
15.	Gawel M, Zalewska E, Szmidt-Sałkowska E, Kowalski J. The value of quantitative EEG in differential diagnosis of Alzheimer's disease and subcortical vascular dementia. J Neurol Sci. 2009;283(1-2):127-133.	Not focused on PD.
16.	Liedorp M, van der Flier WM, Hoogervorst EL, Scheltens P, Stam CJ. Associations between patterns of EEG abnormalities and diagnosis in a large memory clinic cohort. Dement Geriatr Cogn Disord. 2009;27(1):18-23.	Not focused on QEEG.
17.	Morita A, Kamei S, Serizawa K, Mizutani T. The relationship between slowing EEGs and the progression of Parkinson's disease. J Clin Neurophysiol. 2009;26(6):426-429.	Not focused on cognitive states.
18.	Bonanni L, Franciotti R, Onofrj V, Anzellotti F, Mancino E, Monaco D, Gambi F, Manzoli L, Thomas A, Onofrj M. Revisiting P300 cognitive studies for dementia diagnosis: Early dementia with Lewy bodies (DLB) and Alzheimer disease (AD). Neurophysiol Clin. 2010;40(5-6):255-265.	Not focused on PD.
19.	Schlede N, Zimmermann R, Ehrensperger MM, Gschwandtner U, Hardmeier M, Hatz F, Monsch AU, Naegelin Y, Fuhr P. Clinical EEG in cognitively impaired patients with Parkinson's Disease. J Neurol Sci. 2011;310(1-2):75-78	Not focused on QEEG.
20.	Moretti DV, Zanetti O, Binetti G, and Frisoni GB. Quantitative EEG Markers in Mild Cognitive Impairment: Degenerative versus Vascular Brain Impairment. Int J Alzheimers Dis. 2012;2012:917537. doi: 10.1155/2012/917537.	Not focused on PD.
21.	Snaedal J, Johannesson GH, Gudmundsson TE, Blin NP, Emilsdottir AL, Einarsson B, Johnsen K. Diagnostic accuracy of statistical pattern recognition of electroencephalogram registration in evaluation of cognitive impairment and dementia. Dement Geriatr Cogn Disord. 2012;34(1):51-60.	Not focused on PD (patients with PD dementia and dementia with Lewy bodies were combined).
22.	George JS, Strunk J, Mak-McCully R, Houser M, Poizner H, Aron AR. Dopaminergic therapy in Parkinson's disease decreases cortical beta band coherence in the resting state and increases cortical beta band power during executive control. Neuroimage Clin. 2013;3:261-270.	Not focused on cognitive states.
23.	Han CX, Wang J, Yi GS, Che YQ. Investigation of EEG abnormalities in the early stage of Parkinson's disease. Cogn Neurodyn. 2013;7(4):351-359.	Not focused on cognitive states.

24.	Lainscsek C, Hernandez ME, Weyhenmeyer J, Sejnowski TJ, Poizner H. Non-linear dynamical analysis of EEG time series distinguishes patients with Parkinson’s disease from healthy individuals. Front Neurol. 2013;4:200.	Not focused on cognitive states.
25.	Heinrichs-Graham E, Kurz MJ, Becker KM, Santamaria PM, Gendelman HE, Wilson TW. Hypersynchrony despite pathologically reduced beta oscillations in patients with Parkinson's disease: a pharmaco-magnetoencephalography study. J Neurophysiol. 2014;112(7):1739-1747.	Not focused on cognitive states.
26.	Herz DM, Siebner HR, Hulme OJ, Florin E, Christensen MS, Timmermann L. Levodopa reinstates connectivity from prefrontal to premotor cortex during externally paced movement in Parkinson's disease. Neuroimage. 2014;90:15-23.	Not focused on cognitive states.
27.	Melgari JM, Curcio G, Mastrolilli F, Salomone G, Trotta L, Tombini M, di Biase L, Scrascia F, Fini R, Fabrizio E, Rossini PM, Vernieri F. Alpha and beta EEG power reflects L-dopa acute administration in parkinsonian patients. Front Aging Neurosci. 2014;6:302	Not focused on cognitive states.
28.	Yuvaraj R, Murugappan M, Ibrahim NM, Sundaraj K, Omar MI, Mohamad K, Palaniappan R, Satiyan M. Inter-hemispheric EEG coherence analysis in Parkinson's disease: assessing brain activity during emotion processing. J Neural Transm. 2015;122(2):237-252.	Not focused on cognitive states.
29.	Yuvaraj R, Murugappan M, Ibrahim NM, Omar MI, Sundaraj K, Mohamad K, Palaniappan R, Satiyan M. Emotion classification in Parkinson's disease by higher-order spectra and power spectrum features using EEG signals: a comparative study. J Integr Neurosci. 2014;13(1):89-120.	Not focused on cognitive states.
30.	Yuvaraj R, Murugappan M, Omar MI, Ibrahim NM, Sundaraj K, Mohamad K, Satiyan M. Emotion processing in Parkinson's disease: an EEG spectral power study. Int J Neurosci. 2014;124(7):491-502.	Not focused on cognitive states.
31.	Benz N, Hatz F, Bousleiman H, Ehrensperger MM, Gschwandtner U, Hardmeier M, Ruegg S, Schindler C, Zimmermann R, Monsch AU, Fuhr P. Slowing of EEG background activity in Parkinson's and Alzheimer's disease with early cognitive dysfunction. Front Aging Neurosci. 2014 Nov 18;6:314.	Not focused on cognitive states.
32.	Bonanni L, Perfetti B, Bifolchetti S, Taylor JP, Franciotti R, Parnetti L, Thomas A, Onofrj M. Quantitative electroencephalogram utility in predicting conversion of mild cognitive impairment to dementia with Lewy bodies. Neurobiol Aging. 2015;36(1):434-445.	Not focused on PD.
33.	van Dellen E, de Waal H, van der Flier WM, Lemstra AW, Slooter AJ, Smits LL, van Straaten EC, Stam CJ, Scheltens P. Loss of EEG Network Efficiency Is Related to Cognitive Impairment in Dementia With Lewy Bodies. Mov Disord. 2015 Jul 16. doi: 10.1002/mds.26309. [Epub ahead of print]	Not focused on PD.
34.	Engedal K, Snaedal J, Hoegh P, Jelic V, Andersen BB, Naik M, Wahlund LO, Oeksengaard AR. Quantitative EEG applying the statistical recognition pattern method: a useful tool in dementia diagnostic workup. Dement Geriatr Cogn Disord 2015;40:1-12.	Not focused on PD.
35.	Markser A, Maier F, Lewis CJ, Dembek TA, Pedrosa D, Eggers C, Timmermann L, Kalbe E, Fink GR, Burghaus L. Deep brain stimulation and cognitive decline in Parkinson's disease: The predictive value of electroencephalography. J Neurol. 2015 Jul 11. [Epub ahead of print]	DBS patients included.
36.	Mostile G, Nicoletti A, Dibilio V, Luca A, Pappalardo I, Giuliano L, Cicero CE, Sciacca G, Raciti L, Contrafatto D, Bruno E, Sofia V, Zappia M. Electroencephalographic lateralization, clinical correlates and pharmacological response in untreated Parkinson's disease. Parkinsonism Relat Disord. 2015 Aug;21(8):948-953.	Not focused on cognitive states.
37.	Song Y, Zang DW, Jin YY, Wang ZJ, Ni HY, Yin JZ, Ji DX. Background rhythm frequency and theta power of quantitative EEG analysis: predictive biomarkers for cognitive impairment post-cerebral infarcts. Clin EEG Neurosci. 2015;46(2):142-146	Not focused on PD.
38.	Swann NC, de Hemptinne C, Aron AR, Ostrem JL3, Knight RT, Starr PA. Elevated synchrony in Parkinson disease detected with electroencephalography. Ann Neurol. 2015 Aug 20. doi: 10.1002/ana.24507. [Epub ahead of print]	Not focused on cognitive states.
